# Function of Metallothionein-3 in Neuronal Cells: Do Metal Ions Alter Expression Levels of MT3?

**DOI:** 10.3390/ijms18061133

**Published:** 2017-05-25

**Authors:** Jamie Bousleiman, Alexa Pinsky, Sohee Ki, Angela Su, Irina Morozova, Sergey Kalachikov, Amen Wiqas, Rae Silver, Mary Sever, Rachel Narehood Austin

**Affiliations:** 1 Department of Chemistry, Barnard College of Columbia University, New York, NY 10027, USA; jb3424@barnard.edu (J.B.); amp2248@barnard.edu (A.P.); ski@barnard.edu (S.K.); su.angela1@gmail.com (A.S.); 2 Center for Genome Technology and Biomolecular Engineering, Department of Chemical Engineering, Columbia University, New York, NY 10027, USA; im198@columbia.edu (I.M.); sk363@columbia.edu (S.K.); 3 Department of Biology, Barnard College of Columbia University, New York, NY 10027, USA; aw2868@barnard.edu; 4 Department of Psychology and Program in Neuroscience, Barnard College of Columbia University, New York, NY 10027, USA; 5 Department of Psychology, Columbia University, New York, NY 10027, USA; 6 Department of Pathology and Cell Biology Columbia Health Sciences, New York, NY 10027, USA

**Keywords:** metallothionein, MT3, metalloneurochemistry, lead neurotoxicity, gene expression, microarrays, dentate gyrus

## Abstract

A study of factors proposed to affect metallothionein-3 (MT3) function was carried out to elucidate the opaque role MT3 plays in human metalloneurochemistry. Gene expression of *Mt2* and *Mt3* was examined in tissues extracted from the dentate gyrus of mouse brains and in human neuronal cell cultures. The whole-genome gene expression analysis identified significant variations in the mRNA levels of genes associated with zinc homeostasis, including *Mt2* and *Mt3*. *Mt3* was found to be the most differentially expressed gene in the identified groups, pointing to the existence of a factor, not yet identified, that differentially controls *Mt3* expression. To examine the expression of the human metallothioneins in neurons, mRNA levels of *MT3* and *MT2* were compared in BE(2)C and SH-SY5Y cell cultures treated with lead, zinc, cobalt, and lithium. *MT2* was highly upregulated by Zn^2+^ in both cell cultures, while *MT3* was not affected, and no other metal had an effect on either *MT2* or *MT3*.

## 1. Introduction

Mammalian metallothionein-3 isoform (MT3) is an unusual protein with a puzzling role in neurochemistry [1]. It has the hallmarks of a typical mammalian metallothionein and is comprised of 68 amino acids, 20 of which are cysteines. Hence, it is assumed that, like other metallothioneins (MT), it binds essential metal ions such as zinc (Zn) and copper (Cu) and sequesters toxic metal ions such as cadmium (Cd) and silver (Ag) [2]. Most mammalian metallothioneins are induced by high levels of zinc by a mechanism initiated by Zn^2+^ binding to a metal-responsive transcription factor [3]. Because toxic metals such as Cd^2+^ and Ag^+^ displace zinc bound to metallothionein, their presence also indirectly leads to elevated MT levels.

Despite the presence of the canonical 20 cysteines in its structure, MT3 was first identified not as a metallothionein, but as an inhibitor of neuronal growth [4,5]. Although the molecular basis of its ability to inhibit neuronal growth is not understood, the effect has been connected to the seven-amino-acid acidic region that is unique to MT3 [6]. Recently, MT3 has been shown to bind to β-actin and facilitate actin polymerization [7]. β-Actin polymerization is involved in cytoskeletal growth, fueling speculation that the neuronal growth inhibitor effects of MT3 could occur via an interaction with β-actin [8].

MT3 is specifically expressed in the normal human brain and is significantly under-expressed in the brains of patients with Alzheimer’s disease [1,4,9]. Little is known about what constitutes “normal” bioinorganic chemistry in the central nervous system [10], though recent works point to essential roles for zinc and copper ions in the brain and suggest that both ions can function in neuronal signaling [11]. Therefore, it is noteworthy that MT3 might modulate the bioinorganic chemistry of essential (zinc, copper), therapeutic (lithium) and toxic (lead) metal ions in the brain. MT3 has been speculated to play a special role in mediating the chemistry of copper, which has been found in the brain not only as bound to biomolecules and highly regulated, but also as existing in dynamic, loosely bound pools [11]. We have speculated that MT3 might be involved in the effect that the exposure to lead (Pb) has on brain development [12]. It is well known that exposure to lead during childhood leads to irreversible long-lasting developmental effects, including perhaps an increased risk of certain forms of mental illnesses [13,14]. Based on a report that lithium chloride treatment leads to decreased levels of *Mt3* in mice brains, we have considered the possibility that MT3 might be involved in the pharmacological benefits of lithium treatment for people suffering from bipolar mood disorder [15].

In this paper, we explore factors that may potentially impact MT3 gene expression and, indirectly, function. MT3 is known to be abundant in the dentate gyrus (DG) [16]. Our study of gene expression in the DG of a population of litter-mate matched mice, presented here, revealed significant differences between tested mice in the expression levels of *Mt3*, along with a suite of other genes associated with zinc homeostasis. Significantly, *Mt2* was upregulated in one population of mice, while *Mt3* was upregulated in the other population of mice, pointing to a very different mechanism of regulation of these two metallothioneins.

We also examine some inorganic factors that may have an impact on expression levels of metallothioneins in human neuronal cells. There is conflicting published data about the role that metal ions have on the *MT3* levels, pointing to the importance of studying *MT3* expression in multiple cell lines [17,18,19]. We show that, compared to *MT2*, *MT3* responds differently to identical metal treatments. The effects that metal ions have on *MT3* levels are far more modest than the effects that these metal ions have on *MT2* levels. This fact, again, is pointing to a possible regulatory mechanism that differentially controls the expression of *MT2* vs. *MT3* in neuronal tissues.

## 2. Results

This study focused on gaining a better understanding of MT3 metalloneurochemistry by examining relationships between *Mt3/Mt2* and known zinc homeostasis gene expression in mice brains and by comparing how *MT3* and *MT2* levels in neuronal cell cultures respond to metal ion treatments. Ultimately, this work is part of ongoing efforts in our laboratories to connect molecular-level knowledge about the metal-mediated processes in which MT3 participates and its more systemic role in mammal neurobiology.

### 2.1. Analysis of Expression of Zinc Homeostasis Genes in Mice Dentate Gyrus

The expression profiles of genes related to Zn homeostasis were assessed using data mining in a whole-genome gene expression dataset that was produced using DG tissues extracted from adult mice brain samples. The dataset was generated by microarray analysis of the mouse gene complement represented by 60,000 probes on Agilent gene expression arrays and consisted of 21,000 genes that were identified as present based on their hybridization signals above the background.

**Figure 1:**
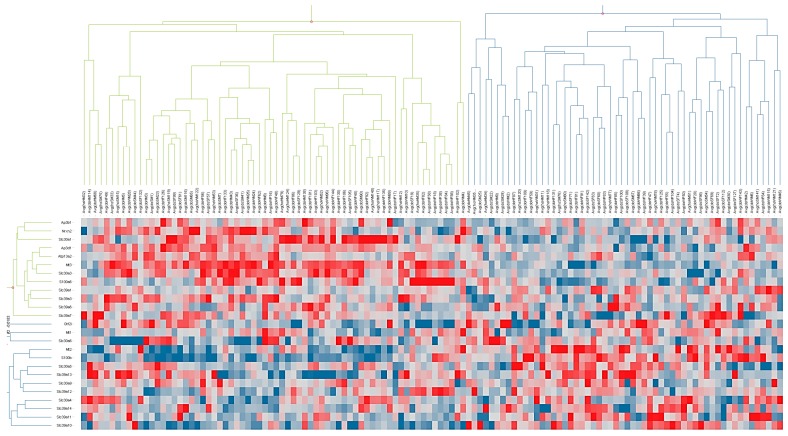
Hierarchical clustering of gene expression profiles related to the expression of Zn transporters. Genes coding for Zn transporters are shown as rows, and individually tested animal samples are shown as columns. The expression changes in the heat map are represented by z-scores that are color-coded according to their values: red squares correspond to positive changes in expression levels (upregulation); blue squares correspond to negative changes in gene expression (downregulation); and white represents the absence of changes. Hierarchical clustering of the gene expression profiles revealed the presence of two distinct groups of animals, shown as green and blue trees, respectively, on the top of the figure.

Gene Ontology search for cellular zinc ion homeostasis and related terms revealed 39 genes, 25 of which were found to be expressed in DG based on our microarray dataset (Table 1). Among these genes, there were three that code for metallothionein proteins related to Zn-ion transport and all the major zinc transporters from the Solute Carrier Protein families 30 and 39. Using hierarchical clustering, a search for possible patterns in expression of these genes in the DG tissue samples revealed a presence of two groups of animals. Nearly equal in size, these two groups can be identified by their group-specific co-expression patterns of Zn-binding transporters (Figure 1).

**Figure 2:**
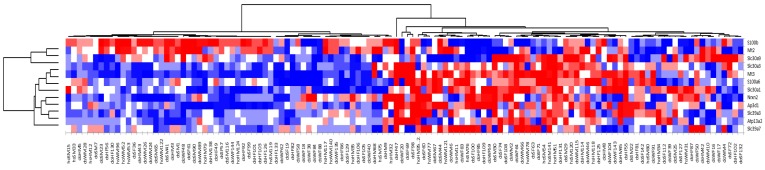
Gene expression profile clustering based on the 11 genes that differentiate animal groups A and B. Genes are shown as rows, and animals (dentate gyrus samples) are shown as columns. The expression changes in the heat map are represented by *z*-scores that are color-coded according to their values: red squares correspond to positive changes in expression levels (upregulation); blue squares correspond to negative changes in gene expression (downregulation); and white represents the absence of changes.

To determine the specific Zn transporters whose expression drives the separation between the two animal groups, we performed a *t*-test analysis of gene expression between these groups. The *t*-test analysis was followed by false discovery rate (FDR) correction using the Benjamini-Hochberg procedure. The analysis revealed 11 genes coding for metallothioneins and transporters (shown in color in Table 2) as having a major impact on the segregation of the animal samples into two groups. Re-clustering the expression profiles of the samples based on these 11 genes (Figure 2) revealed two groups, denoted Groups A and B. Group A is characterized by having the genes coding for *Mt2* and *S100b* (Ca-binding protein B) upregulated. The remaining 9 of the 11 discriminating genes were upregulated in Group B, with *Mt3* showing the largest difference in its expression level when compared to that in Group A.

To find other genes and molecular functions that distinguish Group A from Group B, in addition to those involved in Zn-ion transport, we performed a global (genome-wide) comparison of gene expression between the two groups and analyzed functional enrichment of the genes that are expressed differently in Group A vs. Group B. We found that both groups had a large number of over-expressed genes coding for metal-binding proteins, but those genes that were found to be upregulated in Group A were different from those upregulated in Group B. In particular, about 250 of such genes were upregulated in Group A, while 237 different genes were upregulated in Group B (Table S1). In addition, Group A had a significant enrichment in genes related to synaptic function and signal transduction pathways.

The gene set that was upregulated in Group B was marked with statistically significant upregulation. These genes are enriched in functional categories related to ribosomal function, active transcription and translation, and other processes characteristic of metabolically active cells. Remarkably, in this group, several large subsets of upregulated genes are disease-related, and include genes involved in functional pathways associated with Huntington’s, Parkinson’s, and Alzheimer’s diseases. The common regulatory themes governing these differentially expressed genes in the dentate gyrus of the two groups of mice still remain to be found. It is also noteworthy that these differences are not related to differences in sex.

### 2.2. Analysis of MT2 and MT3 mRNA Expression after Metal Treatment

In order to measure the relative expression of MT2/3 mRNA in cells exposed to various excess metal treatments, RNA was extracted and reverse transcribed to yield a cDNA library, followed by qPCR-based determination of relative gene expression levels. While different time points were explored (data not shown), the 4 h post-exposure results were used for this study. Table 3 and Table 4 present the change in MT2 and MT3 expression relative to non-metal treated controls and normalized to the control gene *HSP90ABI*. Based on the qPCR data, MT2, in both BE(2)C and SHSY5Y cell lines, was highly upregulated in the presence of 100 μM Zn^2+^. The data also show, with equal clarity, that 100 μM Zn^2+^ had no effect on MT3 levels in either cell line. No other metal had a significant effect on either MT2 or MT3 levels in these cell lines (data from all experiments shown in Figure S1). Preliminary studies indicated that Cu(II) did not have a statistically significant effect on MT3 expression.

## 3. Discussion

Metallothioneins are small proteins whose molecular simplicity belies a history replete with efforts to definitively determine their function [20,21,22]. As a member of this protein family known for its confusing biochemical functions, MT3 plays an even more opaque role in mammalian biology. The presented work strives to provide an insight into the biology of MT3 by studying the factors that regulate its expression as well as characterizing the degree of structural changes conferred by the binding of the protein with different metal ions.

Metallothioneins are thought to act as zinc chaperones transporting zinc ions to their target locations and perhaps facilitating their release when warranted [1]. Consistent with this function, most metallothioneins are upregulated by high levels of zinc. The presence of a metal response element (MRE) in the promoter region of *MT1* and *MT2* causes most metallothioneins to be induced by zinc [3]. The MRE is activated via interaction with the zinc finger-containing metal transcription factor 1 (MTF1) [23]. MTF1 is responsible for regulating a number of genes associated with heavy metals and oxidative stress [23]. Metallothionein is the prototypical gene target for MTF1.

Metallothioneins are also thought to function as “heavy metal sponges,” binding toxic metal ions and potentially sequestering them. Heavy metals, including cadmium and silver, have been repeatedly shown to increase metallothionein levels. However, the effect is an indirect one. Heavy metals affect MT levels because they displace zinc from metallothionein, and the displaced zinc binds to the metal transcription factor, which in turn binds to the MRE and activates the transcription of metallothioneins [24]. Interestingly, despite the fact that lead is a heavy metal, it has never been clear whether lead affects the levels of any metallothioneins [25,26,27]. We recently showed that lead can displace zinc from Mt3 with an equilibrium constant of about 1 × 10^4^ and thus should be able to displace zinc in vivo, leading to the upregulation of MTs by the mechanism described above [12]. Nevertheless, some published studies show no change in MT levels following lead treatment [17], while others do show a change [28].

Metallothioneins have also long been associated with a role in ameliorating oxidative stress [22]. MTs can release Zn^2+^ and two electrons while forming a disulfide bond. This may be relevant to immune responses and inflammation [22,29]. MTF1 also binds to the metal response element of other genes involved in cellular redox homeostasis, including selenoproteins H and W [30,31]. Thus, it is speculated that metallothioneins and other proteins involved in combatting oxidative stress will be simultaneously overexpressed under conditions of oxidative stress.

Very significantly, however, *MT3* appears to be regulated differently from other mammalian metallothioneins [18,32]. *MT3* does not appear to respond to zinc levels in either astrocytes or neurons in the same way that other MTs respond to zinc, even though the promoter region of *MT3* has several regions that are similar to the metal response elements that regulate *MT1* and *MT2* levels [1,5,17,19,32]. MTF1 does not bind to the MRE sequence in the *MT3* promoter region [32].

Both our cell culture data and our microarray data are consistent with the assertion that, in the brain, *MT3* is regulated differently from other metallothioneins. Our cell culture data shows that, in two different neuronal cell lines, addition of zinc leads to a significant overexpression of *MT2*, while having essentially no effect on *MT3*.

The microarray data demonstrate that, among a group of littermate-matched mice, approximately half the population (Group A) had statistically significant upregulation of *Mt2* and *S100b*, while the other half of the population (Group B) had statistically significant upregulation of *Mt3* and a number of known zinc transporters (*S100a6*, *Slc30a1*, *Slc39a7*, *Slc30a3*, *Slc39a3*, and *Ap3d1*), as well as *Nrx2* and *Atp13a2*. The fact that *Mt2* was highly expressed in Group A while *Mt3* was highly expressed in Group B indicates that these two genes are regulated differently. Furthermore, the observation that *Mt3* was upregulated along with a number of other zinc transporters suggests that this protein has a role in zinc homeostasis in the brain.

Of particular note is the differential pattern of coexpression of members of the S100 family. *S100b*, which was upregulated together with *Mt2*, expresses a calcium- and zinc-binding protein involved in immune responses. *S100a6*, which is upregulated with Mt3, expressed a calcium- and zinc-binding protein that has been associated with misregulation of zinc levels in several neurodegenerative diseases [33]. *S100a6* is overexpressed in mouse models of amyotrophic lateral sclerosis (ALS) and Alzheimer’s disease and is speculated to contribute to disease progression via its high affinity for zinc [34]. It has also been associated with cell proliferation and neuronal degeneration, processes with which *MT3* is also associated [35,36,37,38]. Other researchers have noted that *S100b* and *S100A6* are differentially expressed in human cortical tissue and have speculated that despite being members of the same family of enzymes they play different neurochemical roles [38]. *S100b* has been linked to neuronal growth, including the formation of neurite extension [38], while *S100a6* has been associated with cytoskeletal dynamics (as has *MT3* [7,8]) [39]. *S100a6* is known to be upregulated under conditions of oxidative stress [39]. *S100b* has been associated with the protective effects of nutritional zinc against methyl mercury exposure [40], as well as linked to *MT2* expression in bipolar mood disorder [41]. Additional studies are underway in our laboratories to further explore the molecular mechanisms that link this differential expression of *MT2*/*S100b* and *MT3*/*S100A6*.

There are a few factors that have been reported to affect *MT3* expression. These include lithium (downregulation), age (upregulation) and hypoxia (upregulation), but in each case, the molecular mechanism for this regulation is not understood. Lithium was shown to decrease *MT3* expression in mice that were given lithium chloride [15]. Our cell culture results, however, showed essentially no effect of lithium on *MT3* levels. The antidepressant eugenol has also been shown to induce *Mt3* expression in the hippocampus of mice while having no effect on *Mt1* levels [42]. Both of these experiments further suggest that MT3 may play a role in aspects of brain chemistry not normally associated with metallothionein function and also point to regulation of MT3 by a non-MRE pathway.

MT3 levels increase with age [36,43,44,45,46]. In mice brains, Mt3 was found in higher concentrations after 12 weeks of age [43]. In rat brains, a very large increase in Mt3 levels was seen when the rats passed middle age (16 months) [36]. The authors speculate that, because MT3 has more Cu-thionein character, its upregulation could indicate an enhanced role in copper chelation to protect the aging brain from copper redox toxicity. However, it is unclear by what mechanism aging could lead to higher MT3 levels [36].

MT3 also responds to hypoxia [45,46,47]. In one study of hypoxia, *MT3* was the gene most sensitive to hypoxia, with 3- and 7-fold increases in expression levels after 10 and 30 min treatments, respectively [45]. In another study, *MT3* levels increased by a factor of more than 600 in 60 min upon exposure to hypoxic conditions [46]. MT3 gene expression can also reportedly be substantially induced by hypoxia mimetics (CoCl_2_, desferrioxamine, dimethyloxalylglycine), which indicates that it is transcriptionally regulated through HIF1 [46]. Hypoxia has been suggestively linked to an imbalance in zinc homeostasis [47]. Also, while other metallothioneins have been shown to respond to hypoxia, in the case of other MTs, activation via the MRE was implicated [48]. Again, in our cell culture experiments, the hypoxia mimic Co^2+^ showed essentially no effect on either *MT2* or *MT3* expression levels.

It is challenging to study factors that influence *MT3* levels, and our work illustrates this as well. Few established cell lines express *MT3* to levels comparable to the levels that have been measured in the brain [31]. In this study, we selected two different neuronal cell lines, both of which were known to have measurable but not extremely high *MT3* levels, making it possible to study treatments that might lead to increased (or decreased) gene and protein expression. Even still, the cell culture work does not appear to capture all of the complexity visible in animal models.

Considering that MT3 is in so many respects similar to MT1 and MT2, how can one explain its strikingly different neurochemistry? Part of the answer presumably rests with the small structural differences between MT3 and other MTs [49]. The unique N-terminal sequence of MT3 is required for the growth inhibitory activity of MT3 in the neural system [50]. It has also been shown to be responsible for Cd^2+^-induced death of the proximal tubule cell [50]. These amino acids have also been shown to be responsible for zinc-specific MT3 binding to actin [7]. Protein–protein interactions, modulated by MT3’s unique structure, may be critical to many of MT3’s neurochemical functions [9,49]. The 3D structure of metallothionein is determined by metal binding. Thioneins are unstructured and unfolded in the absence of metal ions and then fold into bilobal proteins upon binding seven divalent metal ions [51,52]. Suprametalation has been suggested to cause the protein to rearrange to form a single, more globular structure [53]. Apo-MT2 and apo-MT3 in solution have practically identical structures with low helical content [54], supporting the common notion that apo-metallothioneins exhibit little or no secondary structure [55]. In the presence of metals, however, metallothioneins fold into varied structures. Metallothioneins bind divalent metals such as Zn^2+^ and Cd^2+^ in tetrahedrally coordinated M_3_S_9_ and M_4_S_11_ binding clusters, separated with sections of random coil. These structures are characterized by circular dichroism spectra that show a positive peak at 240 nm [56].

We and others have speculated that some of the neurotoxic effects of lead exposure might be mediated by differences in metallothionein structure when bound to lead vs. zinc [12,57,58]. Zinc prefers a coordination number of four, corroborated by its tetrahedral coordination with thiol sites [56]. In contrast to the coordination chemistry of Zn^2+^, that of Pb^2+^ is much more complex and varied [59,60]. Recent evidence indicates few four-coordinate Pb(II) complexes; they are either three-coordinate or five-coordinate [31]. Possibly, lead’s (potentially) stereochemically active lone pair plays a role in determining its geometric preferences [30,32]. He et al. speculate that lead forms a trigonal pyramidal coordination when bound to MT2 with three sulfurs and electron donating oxygens (Pb–S_3_O) [57]. The known differences in metal binding preferences between MT3 and other metallothioneins, including the presence of an 8th metal binding site in MT2, is likely to effect the overall structure of metal-replete MT3 [54,61,62]. Work is ongoing to determine if these structural differences lead to functional differences in the way that MT3 interacts with partner proteins [9].

Part of the answer also presumably lies with the differential expression of MT3. This work confirms the differential expression of MT3 but does not elucidate what the molecular mechanism is by which it is regulated. Additional work in our labs is also underway to test hypotheses associated with regulatory pathways for MT3.

## 4. Materials and Methods

### 4.1. Materials

Metal salts used in this work include: lead acetate (99–103%, Thermo Fisher Scientific, Waltham, MA, USA), zinc sulfate monohydrate (99%, Strem Chemicals, Newburyport, MA, USA), cobalt (II) chloride hexahydrate (98.0–102.0%, Sigma Aldrich, St. Louis, MO, USA), and lithium sulfate monohydrate (99%, Sigma Aldrich).

An ÄKTA Pure 25 L FPLC (GE Healthcare, Pittsburgh, PA, USA) was used for protein purification, and a Forma Anaerobic System 1025 (Thermo Forma, Marietta, OH, USA) was used for anaerobic protein manipulations. Low-speed centrifugation was done with an Allegra X-30R benchtop centrifuge (Beckman Coulter, Indianapolis, IN, USA) and higher speed centrifugation above 10,000 rpm was done with an Avanti J-265 XPI superspeed centrifuge (Beckman Coulter). Sonication was done with a Vibra-Cell VC 505 Ultrasonic Liquid Processor (Sonics & Materials, Inc., Newtown, CT, USA), and lyophilization was done on a FreeZone 2.5 Liter Benchtop Freeze Dry System (Labconco, Kansas City, MO, USA). UV-Vis spectra were taken on a Varian Cary 50 UV-Vis spectrometer (Agilent Technologies, Santa Clara, CA USA).

MES, MOPS, and bis-Tris buffers were purchased in highest purity from Sigma Aldrich (St. Louis, MO, USA), as were metal salts, dithiothreitol (DTT), and 5,5′-dithiobis-(2-nitrobenzoi acid) (DTNB). Tris(2-carboxyethyl)phosphine hydrochloride (TCEP-HCl) was purchased from Thermo Fisher Scientific (Waltham, MA, USA). Thrombin from bovine plasma was obtained from GE Healthcare Life Sciences (Pittsburgh, PA, USA). GSTrap FF columns, HiTrap desalting columns, and a HiLoad 26/600 75 pg size exclusion column were also purchased from GE Healthcare Life Sciences (Pittsburgh, PA, USA). All buffers were prepared with milli-Q (18.2 MΩ·cm) water, filtered through 0.22 μm filter unit, and degassed under vacuum until no air bubbles were seen. Metal stock solutions were prepared using the appropriate mass of metal salt and then diluted with oxygen-free buffers in the anaerobic chamber.

### 4.2. Animal Studies Ethics Statement and Animal Use

All animal care and testing protocols were approved by the Institutional Animal Care and Use Committee at Columbia University, animal welfare assurance # A3007-01 (31 July 2013).

C57BL/6 mice were purchased from Jackson Laboratories (Bar Harbor, ME, USA) and bred to establish colonies at the Columbia University animal facility (New York City, NY, USA). All mice were group-housed according to institutional guidelines, with water and a commercial mouse diet available ad libitum. Gene expression analysis was performed on age- and sex-matched mice (8- to 12-week-old; 61 females, 63 males).

### 4.3. Tissue Preparation

The dentate gyrus (DG) was dissected from adult mice hippocampi under a stereomicroscope using an established method [63]. Brains were collected from deeply anesthetized mice and placed into ice-cold phosphate-buffered saline (PBS). After exposing the medial side of the brain, the DG was isolated and retrieved using forceps. DG tissue was placed directly into cryovials containing RNAlater and kept at 4 °C overnight. The following day, RNAlater was removed, and samples were frozen at −80 °C until further use.

### 4.4. RNA Extraction, Amplification, and Gene Expression Microarray Experiments

Before tissue homogenization, all surfaces and instruments were wiped clean with an Ambion RNaseZap RNase decontamination solution (Thermo Fisher Scientific). RNA was extracted from DG tissue according to RNeasy Mini Kit instructions (Qiagen, Germantown, MD, USA). DG tissues of individual mice were homogenized using a Powergen 700 homogenizer (Thermo Fisher Scientific) in the kit lysis buffer. Samples were quantified spectrophotometrically using a NanoDrop 2000 (Thermo Fisher Scientific) and further analyzed using a 2100 Bioanalyzer (Agilent Technologies).

10 ng of the extracted RNA were amplified using an Ambion MessageAmp II aRNA Amplification Kit in vitro transcription protocol following the manufacturer instructions (Thermo Fisher Scientific). The resulting aRNA was purified using an Ambion MEGAclear Transcription Clean-Up Kit (Thermo Fisher Scientific), and 2.5 µg of aRNA from each sample were labeled with AlexaFluor647 or AlexaFluor536 using RNA alkylation with monoreactive cisplatin dye complexes from an Invitrogen ULYSIS Nucleic Acid Labeling Kits (Molecular Probes, Thermo Fisher Scientific). To correct for dye-related sensitivity bias in the downstream detection of hybridization signals, the dye selection was balanced between the sample groups across the experiment. Using spectroscopic measurements of the labeled probes, labeling efficiencies were estimated as 0.5–1.5% of the dye with respect to RNA bases. The labeled aRNA samples were hybridized to SurePrint G3 Mouse GE 8x60K Microarray Kit (Agilent) following the manufacturer instructions. After hybridization, the microarray slides were scanned on the two channels using Agilent C scanner (Agilent), and the image segmentation, gridding, and feature extraction were performed using the onboard Scan Control software (v.8.5.1).

### 4.5. Analysis of Microarray Data

The raw hybridization signal intensities were normalized within the arrays by Agilent software following global normalization using the loess function from the Bioconductor package affy (v.1.42.3) [64,65]. Background filtering was performed on Agilent’s “Well Above Background” flag function using score modulation between the relevant groups of the samples. For a gene to be scored as present, the gene expression signals must be flagged “Well Above Background” in more than 50% of samples within a group. Gene function assignments were done using Gene Ontology (AmiGO, v.2.4.26) [66]. The resulting data were imported into the Spotfire (v.7.6.1) for Functional Genomics Suite (TIBCO, New York City, NY, USA) for data visualization and further review using PCA and hierarchical clustering. Analysis of differential gene expression was performed using a *t*-test followed by Benjamini-Hochberg multiple testing correction [67]. Differentially expressed genes for functional enrichment surveys were selected at FDR < 0.05 and fold change of 1.3. Functional enrichment analysis in differentially expressed genes was carried out with DAVID (v.6.8) [68].

### 4.6. General Cell Culture and Metal Treatment

Human neuroblastoma cell lines, BE(2)C and SH-SY5Y (ATCC), were cultured in 1X Dulbecco’s Modified Eagle Media (DMEM) with 4.5 g/L glucose and l-glutamine without sodium pyruvate (Corning Inc., Corning, NY, USA), supplemented with 10% fetal bovine serum (FBS, Thermo Fisher Scientific) using standard mammalian cell culture procedures. Cells were maintained at 37 °C and 5% CO_2_ in a humidified incubator.

One day prior to metal treatment, BE(2)C and SH-SY5Y cells were seeded at 400,000 BE(2)C cells or 450,000 SH-SY5Y cells per well onto tissue culture treated 6-well plates (2 mL media per well). At designated treatment times, the cell culture media was replaced with 2 mL of fresh DMEM with 10% FBS and 100 mM metal solutions of lead acetate, zinc sulfate, cobalt chloride, or lithium sulfate were added to the media to a final concentration of 100 µM metal ion.

### 4.7. Total RNA Extraction, cDNA Synthesis, and qPCR

Prior to RNA extraction, metal containing media was aspirated from the cells and the cells were washed with PBS. Total RNA was extracted using the PureLinkTM RNA Mini Kit (Invitrogen) according to manufacturer’s instructions for monolayer cells. The quantity and relative purity of the total RNA was measured by UV-Vis spectroscopy on a Nanodrop 2000 spectrophotometer using the absorbance at 260 nm and the 260/280 nm ratio, respectively. All RNA were stored at −80 °C until further use.

Total cellular messenger RNA were reverse transcribed using the First Strand cDNA Synthesis (Quick Protocol, New England BioLabs, Ipswich, MA, USA). Within each trial, total RNA used for reverse transcription were maximized and standardized within the range of 200–1000 ng of RNA. To amplify only the mRNA transcripts, an anchored poly-DT primer (5 mg/mL, IDT DNA) was used. Complementary DNA (cDNA) synthesis was performed in a thermocycler (Bio-Rad, Hercules, CA, USA) according to the following protocol: 42 °C for 60 min, 80 °C for 10 min. The extracted cDNA was incubated at 4 °C for immediate use and stored at −20 °C for future use.

Real time quantitative PCR (qPCR) was performed using PowerUP SYBR Green Master Mix (Thermo Fisher Scientific) according to the manufacturer’s protocol for a 20 µL reaction using 2 µL of the cDNA product as the PCR template. *MT3*, *MT2*, and the housekeeping gene *HSP90AB1* were quantified (in triplicate) for each metal treatment condition as well as the non-treatment control. The primers used for quantitative PCR amplification of *MT2*, *MT3*, and *HSP90AB1* cDNA are listed in Table 5. A LightCycler 480 instrument (Roche Molecular Diagnostics, Pleasanton, CA, USA) was used for qPCR amplification and monitoring, according to the PowerUp SYBR Green Master Mix thermal cycling protocol guidelines. Based on the primers used, the annealing temperature for the qPCR was set at 58 °C. qPCR data was analyzed using the ΔΔ*C*_P_ method [69]. The presence of MT3 in the cell lines was confirmed by western blot analysis and by SDS-PAGE gels with purified Mt3 as a control.

### 4.8. Protein Purification

Recombinant mouse Zn_7_-Mt3 was purified by following a modified protocol from our previous study [12,70,71]. Cells were induced at OD_600_ ~ 0.7 by adding 1 mM isopropyl β-d-1-thiogalactopyranoside (IPTG). After incubation at room temperature and 250 rpm for 1 h, ZnCl_2_ was added to a final concentration of 0.5 mM, and the cultures were incubated at 37 °C and 250 rpm for 16–18 h.

Cells were thawed, separated into approximately 15 g portions, and each portion was suspended in a solution of 1.248 g sucrose in 15 mL PBS (140 mM NaCl, 2.7 mM KCl 10 mM Na_2_HPO_4_, 1.8 mM KH_2_PO_4_). Argon was bubbled through the mixture before centrifuging at 8000× *g* for 30 min at 4 °C and collecting the pellets. To a suspension of the pellet in 15 mL PBS, 5.7 mM β-mercaptoethanol (BME) was added. After bubbling with argon, the suspension was centrifuged at 8000× *g* for 30 min at 4 °C. The pellets were collected and resuspended in PBS to a total volume of 15 mL. Just before sonicating the solution for 7 min, with 10 s rest every 5 s, 1 mM DTT and 0.1 mM phenylmetylsulfonyl fluoride (PMSF) were added. The lysed cells were then centrifuged at 8000× *g* for 30 min at 4 °C. The supernatant was collected and centrifuged at 35,000 rpm for 30 min to remove any particulate matter. A Bradford standard assay using bovine serum albumin (BSA) was used to determine the protein concentration of the cell lysate.

Using previously published protocols, GST-tagged Mt3 protein was separated via GSTrap FF columns and cleaved using bovine plasma thrombin [12]. Mt3 was isolated via HiLoad 26/600 75 pg size exclusion column. Mt3 concentration was determined by measuring the absorbance of the samples in 1 mM HCl at 220 nm and calculating using an extinction coefficient of 53,000 M^−1^·cm^−1^ [62]. The presence of Mt3 was confirmed by SDS-PAGE on a 15% polyacrylamide gel, either with silver or Coomassie staining and florescence imaging on bromobimane-modified MT3 samples [72].

Mt3 was concentrated and buffer-exchanged into Milli Q water using 3 kDa Amicon centrifugal filter units. The concentrate was lyophilized overnight, and reconstituted in Tris-HClO_4_ buffer to maintain the pH at 7.0. Mt3 concentration was determined both by using a DTNB assay measuring the concentration of free thiols (20 free thiols per protein) and by measuring the absorbance at 220 nm in 1 mM HCl as described above.

## Figures and Tables

**Table 1:** Genes related to Zn ion homeostasis. Out of the 39 genes listed in Gene Ontology (GO) as associated with Zn ion homeostasis, 25 were expressed above background levels in the hippocampal dentate gyrus samples in this study (shown in red).

Gene	Gene/Product Name	GO Term	Term Description	Registry
*Ap3b1*	adaptor-related protein complex 3, beta 1 subunit	GO:0006829	calcium channel regulator activity	MGI:MGI:4441257|PMID:17349999
*Ap3d1*	adaptor-related protein complex 3, delta 1 subunit	GO:0061088	regulation of sequestering of zinc ion	MGI:MGI:4834177|GO_REF:0000096
*Atp13a2*	ATPase type 13A2	GO:0006882	cellular zinc ion homeostasis	MGI:MGI:4834177|GO_REF:0000096
*Gtf2i*	general transcription factor II I	GO:0051481	cellular zinc ion homeostasis	MGI:MGI:4417868|GO_REF:0000096
*Mt1*	Metallothionein-1	GO:0006882	cellular zinc ion homeostasis	MGI:MGI:2177111|PMID:11792622
*Mt2*	Metallothionein-2	GO:0006882	cellular zinc ion homeostasis	MGI:MGI:2177111|PMID:11792622
*Mt3*	Metallothionein-3	GO:0006882	cellular zinc ion homeostasis	MGI:MGI:5315330|PMID:21359432
*Nrxn2*	neurexin II	GO:0005246	calcium channel regulator activity	MGI:MGI:3040168|PMID:12827191
*S100a6*	ref|Mus musculus S100 calcium binding protein A6 (calcyclin) (S100a6), mRNA [NM_011313]	GO:0005509	calcium ion binding	MGI:MGI:4834177|GO_REF:0000096
*S100b*	ref|Mus musculus S100 protein, beta polypeptide, neural (S100b), mRNA [NM_009115]	GO:0005509	calcium ion binding	MGI:MGI:4417868|GO_REF:0000096
*Slc30a1*	solute carrier family 30 (zinc transporter), member 1	GO:0006882	cellular zinc ion homeostasis	MGI:MGI:3056220|PMID:15452870
*Slc30a3*	solute carrier family 30 (zinc transporter), member 3	GO:0032119	cellular zinc ion homeostasis	MGI:MGI:4441264|PMID:16741752
*Slc30a4*	solute carrier family 30 (zinc transporter), member 4	GO:0061088	regulation of sequestering of zinc ion	MGI:MGI:4459044|GO_REF:0000033
*Slc30a5*	solute carrier family 30 (zinc transporter), member 5	GO:0006882	cellular zinc ion homeostasis	MGI:MGI:4834177|GO_REF:0000096
*Slc30a6*	solute carrier family 30 (zinc transporter), member 6	GO:0061088	regulation of sequestering of zinc ion	MGI:MGI:4459044|GO_REF:0000033
*Slc30a9*	solute carrier family 30 (zinc transporter), member 9	GO:0006829	calcium channel regulator activity	MGI:MGI:1354194|GO_REF:0000004
*Slc39a1*	solute carrier family 39 (zinc transporter), member 1	GO:0006829	calcium channel regulator activity	MGI:MGI:2683827|PMID:14525987
*Slc39a10*	solute carrier family 39 (zinc transporter), member 10	GO:0006882	cellular zinc ion homeostasis	MGI:MGI:5586917|PMID:25074913
*Slc39a11*	solute carrier family 39 (metal ion transporter), member 11	GO:0006829	calcium channel regulator activity	MGI:MGI:1354194|GO_REF:0000004
*Slc39a12*	solute carrier family 39 (zinc transporter), member 12	GO:0006882	cellular zinc ion homeostasis	MGI:MGI:4459044|GO_REF:0000033
*Slc39a13*	solute carrier family 39 (metal ion transporter), member 13	GO:0006882	cellular zinc ion homeostasis	MGI:MGI:4834177|GO_REF:0000096
*Slc39a14*	solute carrier family 39 (zinc transporter), member 14	GO:0006882	cellular zinc ion homeostasis	MGI:MGI:4834177|GO_REF:0000096
*Slc39a3*	solute carrier family 39 (zinc transporter), member 3	GO:0006829	calcium channel regulator activity	MGI:MGI:2683827|PMID:14525987
*Slc39a6*	solute carrier family 39 (metal ion transporter), member 6	GO:0006882	cellular zinc ion homeostasis	MGI:MGI:4834177|GO_REF:0000096
*Slc39a7*	solute carrier family 39 (zinc transporter), member 7	GO:0006882	cellular zinc ion homeostasis	MGI:MGI:4459044|GO_REF:0000033
*Atp7b*	ATPase, Cu++ transporting, beta polypeptide	GO:0006882	cellular zinc ion homeostasis	MGI:MGI:1100407|PMID:9392450
*Cp*	ceruloplasmin	GO:0006879	cellular iron ion homeostasis	MGI:MGI:2152098|GO_REF:0000002
*Heph*	hephaestin	GO:0006879	cellular iron ion homeostasis	MGI:MGI:2152098|GO_REF:0000002
*Kel*	Kell blood group	GO:0006874	cellular calcium ion homeostasis	MGI:MGI:5581641|PMID:23122227
*Pik3c2a*	phosphatidylinositol 3-kinase, C2 domain containing, alpha polypeptide	GO:0071583	zinc II ion transport	MGI:MGI:5543317|PMID:23823722
*Slc30a10*	solute carrier family 30, member 10	GO:0061088	regulation of sequestering of zinc ion	MGI:MGI:4459044|GO_REF:0000033
*Slc30a2*	solute carrier family 30 (zinc transporter), member 2	GO:0061088	regulation of sequestering of zinc ion	MGI:MGI:4459044|GO_REF:0000033
*Slc30a7*	solute carrier family 30 (zinc transporter), member 7	GO:0032119	cellular zinc ion homeostasis	PMID:17720550
*Slc30a8*	solute carrier family 30 (zinc transporter), member 8	GO:0006882	cellular zinc ion homeostasis	MGI:MGI:4417868|GO_REF:0000096
*Slc39a2*	solute carrier family 39 (zinc transporter), member 2	GO:0006829	calcium channel regulator activity	MGI:MGI:2683827|PMID:14525987
*Slc39a4*	solute carrier family 39 (zinc transporter), member 4	GO:0006882	cellular zinc ion homeostasis	MGI:MGI:3028750|PMID:14612438
*Slc39a5*	solute carrier family 39 (metal ion transporter), member 5	GO:0006882	cellular zinc ion homeostasis	MGI:MGI:3522410|PMID:15358787
*Slc39a8*	solute carrier family 39 (metal ion transporter), member 8	GO:0006882	cellular zinc ion homeostasis	MGI:MGI:4459044|GO_REF:0000033
*Slc39a9*	solute carrier family 39 (zinc transporter), member 9	GO:0006829	calcium channel regulator activity	MGI:MGI:1354194|GO_REF:0000004

**Table 2:** Zn transporter genes that differentiate the two identified animal groups. Eleven genes, shown in red and blue, account for the major differences between the two animal groups identified in Figure 1. Other Zn transporter genes do not show statistically significant changes in expression levels between the groups. Among the 11 differentially expressed genes, two (*Mt2* and *S100b*, shown in blue) are upregulated in the animal Group A as compared to Group B.

Genes	*p*-Value	Statistically Significant?	Relative Expression Value, Group A	Relative Expression Value, Group B	log2R
*Ap3d1*	0.000	Yes	10.234	10.015	0.219
*Atp13a2*	0.002	Yes	12.355	12.143	0.212
*Mt2*	0.000	Yes	15.108	15.370	−0.262
*Mt3*	0.000	Yes	15.251	14.301	0.950
*Nrxn2*	0.002	Yes	12.817	12.617	0.200
*S100a6*	0.000	Yes	10.282	9.739	0.542
*Slc30a1*	0.000	Yes	8.177	7.487	0.690
*Slc39a7*	0.023	Yes	11.370	11.198	0.171
*S100b*	0.000	Yes	11.140	11.634	−0.494
*Slc30a3*	0.000	Yes	9.947	9.349	0.598
*Slc39a3*	0.000	Yes	10.368	10.054	0.314
*Slc30a9*	0.162	No	10.367	10.208	0.159
*Gtf2i*	1.000	No	11.863	11.918	−0.055
*Mt1*	0.165	No	7.607	7.404	0.203
*Mt1*	1.000	No	15.846	15.801	0.046
*Slc30a4*	1.000	No	8.711	8.744	−0.033
*Slc30a5*	1.000	No	8.351	8.345	0.006
*Slc30a6*	0.880	No	6.906	6.749	0.157
*Slc30a9*	0.536	No	7.993	7.851	0.143
*Slc39a1*	1.000	No	10.034	10.087	−0.053
*Slc39a10*	0.075	No	9.312	9.489	−0.176
*Slc39a11*	1.000	No	9.436	9.497	−0.061
*Slc39a12*	1.000	No	8.267	8.215	0.053
*Slc39a13*	0.092	No	7.053	7.399	−0.346
*Slc39a14*	1.000	No	8.260	8.351	−0.092
*Slc39a6*	1.000	No	10.298	10.241	0.057

**Table 3:** Relative *MT2* mRNA levels after metal treatment. All results are normalized to non-metal treated control cells and the housekeeping gene *HSP90ABI*. Results are the average of 6 and 5 biological replicates, for BE(2)C and SH-SY5Y cells, respectively. Each biological replicate is the average of 3 technical replicates. Standard errors are presented in parentheses.

Cell Line	No Treatment	Pb	Zn	Co	Li
BE(2)C	1	1.66 (0.77)	24.58 (6.23)	1.45 (0.46)	1.16 (0.37)
SH-SY5Y	1	0.97 (0.04)	16.19 (2.13)	1.18 (0.14)	1.35 (0.27)

**Table 4:** Relative *MT3* mRNA levels after metal treatment. All results are normalized to non-metal treated control cells and the housekeeping gene *HSP90ABI*. Results are the average of 6 and 5 biological replicates, for BE(2)C and SH-SY5Y cells, respectively. Each biological replicate is the average of 3 technical replicates. Standard errors are presented in parentheses.

Cell Line	No Treatment	Pb	Zn	Co	Li
BE(2)C	1	1.30 (0.34)	1.20 (0.44)	1.99 (0.58)	1.16 (0.16)
SH-SY5Y	1	0.66 (0.14)	0.76 (0.28)	1.17 (0.28)	1.23 (0.33)

**Table 5:** Reverse transcription and PCR primers used in this study.

Primer	Sequence	T_m_ (°C)
Anchored Poly DT	5′ TTT TTT TTT TTT TTT TTT TTV N 3′	41.0
*HSP90* Primer 1	5′ TCC TTC TCT CGT TCC TTC TCC 3′	55.7
*HSP90* Primer 2	5′ GTA CCA AAG TGA TCC TCC ATC T 3′	54.0
*MT-2* Forward	5′ CCG ACT CTA GCC GCC TCT T 3′	59.0
*MT-2* Reverse	5′ GTG GAA GTC GCG TTC TTT ACA 3′	55.3
*MT-3* Forward	5′ CTG CGG AGT GTG AGA AGT GT 3′	57.1
*MT-3* Reverse	5′ TTG TCA TTC CTC CAA GGT CA 3′	53.6

## Supplementary Materials

Supplementary materials can be found at www.mdpi.com/1422-0067/18/6/1133/s1.

## Abbreviations

BME	2-Mercaptoethanol
BSA	Bovine serum albumin
DG	Dentate gyrus
DMEM	Dulbecco’s modified eagle medium
DTNB	5,5′-Dithiobis-(2-nitrobenzoi acid)
DTT	Dithiothreitol
FDR	False discovery rate
FPLC	Fast protein liquid chromatography
GST	Glutathione *S*-transferase
MES	2-(*N*-morpholino)ethanesulfonic acid
MOPS	3-(*N*-morpholino)propanesulfonic acid
MRE	Metal regulatory element
MT2	Human metallothionein-2 protein
*MT2*	Human metallothionein-2 gene
MT3	Human metallothionein-3 protein
*MT3*	Human metallothionein-3 gene
Mt2	Mouse metallothionein-2 protein
*Mt2*	Mouse metallothionein-2 gene
Mt3	Mouse metallothionein-3 protein
*Mt3*	Mouse metallothionein-3 gene
MTF1	Metal transcription factor 1
PBS	Phosphate buffer saline
PCA	Principal component analysis
PMSF	Phenylmethylsulfonyl fluoride
qPCR	Quantitative polymerase chain reaction
SDS-PAGE	Sodium dodecyl sulfate polyacrylamide gel electrophoresis
TCEP-HCl	Tris(2-carboxyethyl)phosphine hydrochloride
