# Supplementary material for: Function of Metallothionein-3 in Neuronal Cells: Do Metal Ions Alter Expression Levels of MT3?
Source: Int J Mol Sci. 2017 May 25;18(6):1133. doi: 10.3390/ijms18061133 (PMC5485957; doi:10.3390/ijms18061133)
Supplement: Supplementary file 1 [file ijms-18-01133-s001.pdf]

Supplementary Materials: Function of Metallothionein-3 in Neuronal Cells: Do Metal Ions Alter Expression Levels of MT3?

Jamie Bousleiman, Alexa Pinsky, Sohee Ki, Angela Su, Irina Morozova, Sergey Kalachikov, Amen Wiqas, Rae Silver, Mary Sever, and Rachel Narehood Austin

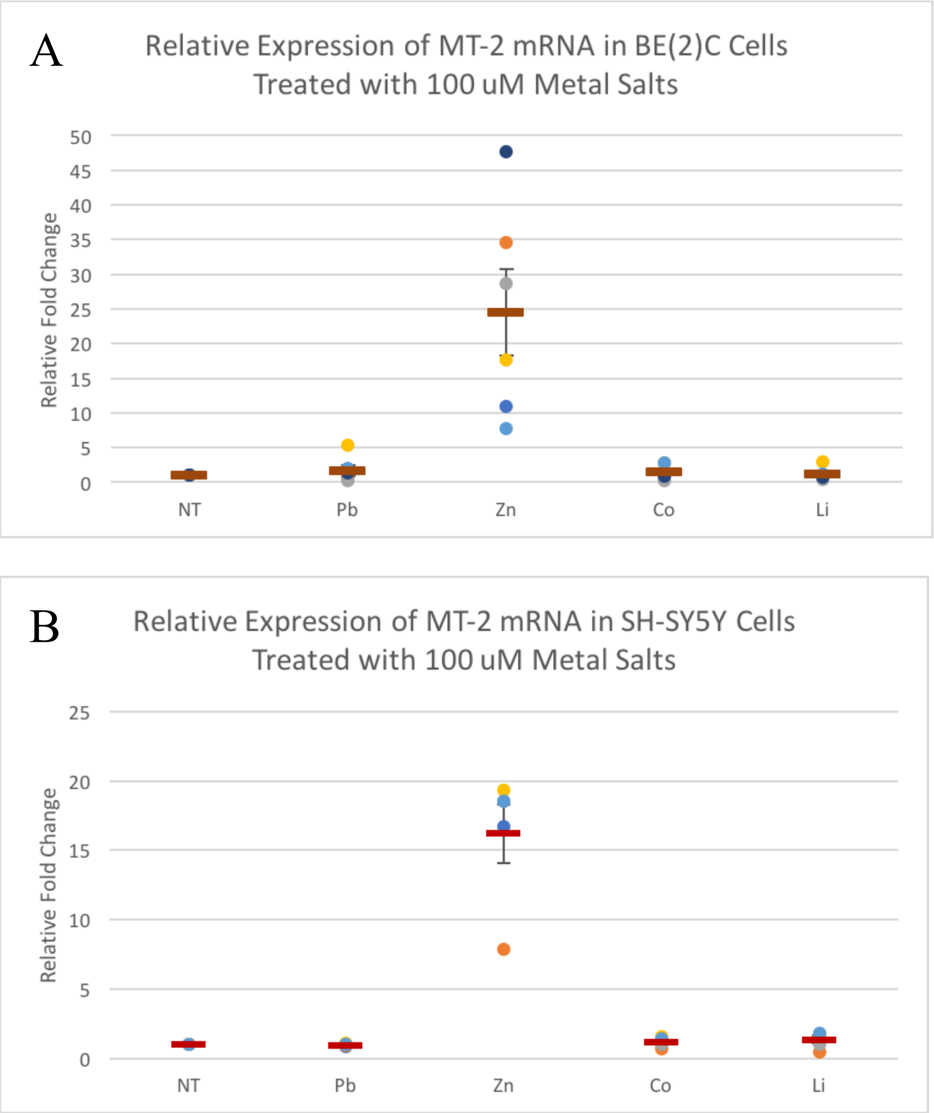

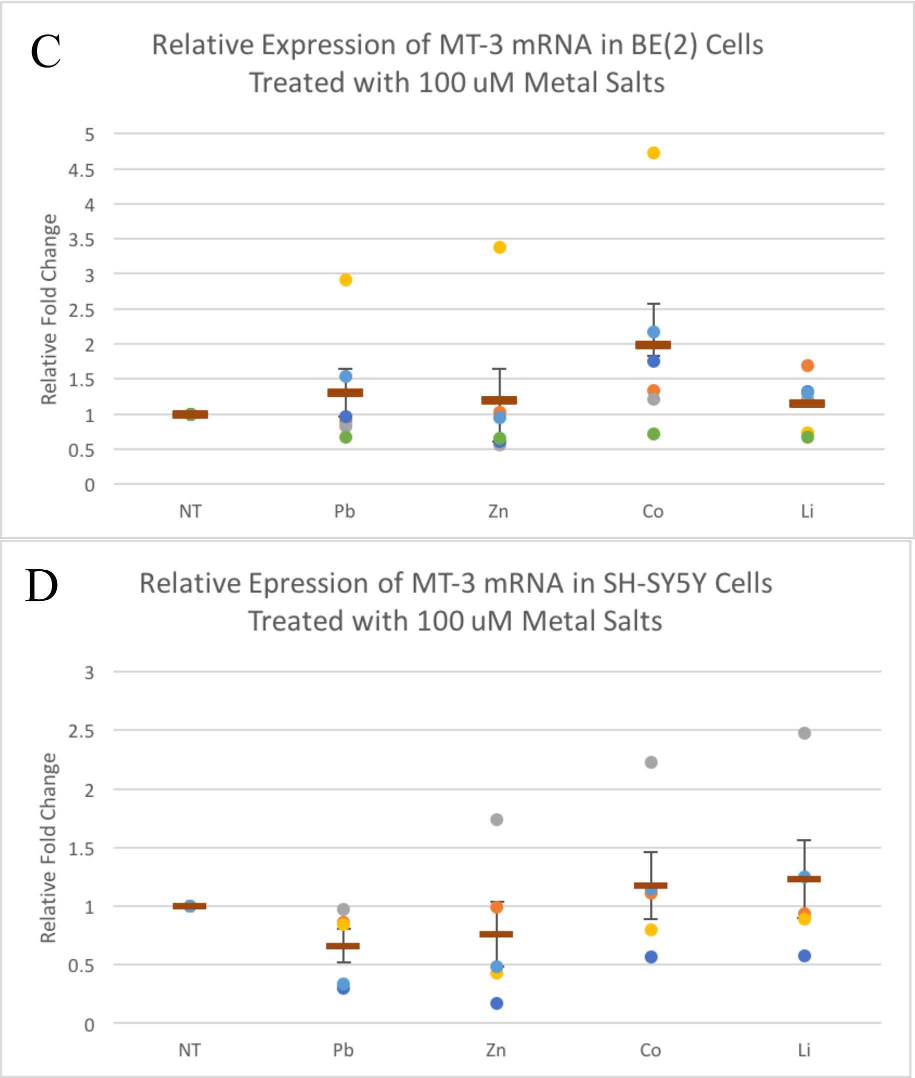

Figure S1. Relative fold change in cDNA expression after various metal treatments. NT = no metal treatment. (A) *MT2* from BE(2)C cells; (B) *MT2* from SH-SY5Y cells; (C) *MT3* from BE(2)C cells; (D) *MT2* from SH-SY5Y cells. Average fold change is shown as a horizontal brown line, and standard deviation is shown as a vertical gray line.

Table S1. Genes for metal-binding and Zn-binding GO categories found among upregulated genes in Group *A* and Group *B*. Enriched terms for *Metal binding*, *Zinc-finger proteins* and *Zinc* were the same for both groups. ***Count*** is the number of differentially expressed genes matching the term; **%** is the percentage of the genes matching the term with respect to all differentially expressed genes that can be functionally annotated for the given group; ***Pval*** is a modified *Fisher Exact* test *P*-value for gene-enrichment analysis estimated using EASE.[1] *P*-values smaller than 0.05 indicate a strong enrichment in the functional annotation term.

	Group A				Group B			
**Term**	**Count**	**%**	**PVal**	**Genes**	**Count**	**%**	**PVal**	**Genes**
**Metal-binding**	248	17	1.24E-05	PNMA3, DZIP1, OVCH2, RP9, ZNRF4, SYT6, RORB, ITSN2, CD209D, BTK, ATP2B1, MAP3K6, MAP3K5, ZFP930, U2AF1, ZRSR1, CDH26, ZFP936, CLCA1, PLD6, RXRG, OLA1, POLB, NUDT10, OGFOD1, PRDM9, PITPNM3, HNF4A, ZGLP1, NEK9, ADAMTS1, UNC13B, FUS, ERMP1, GNAI2, ZCRB1, AKAP13, UBA5, PPAT, PLAGL1, THTPA, MACF1, ZMPSTE24, AEBP2, BMP1, TGFBR1, AGBL4, S100A11, TNNI3, AFG3L1, PPA2, ZFP609, ATP13A4, ZFP940, CBLC, ZFP442, RNF7, HSPB11, RAPSN, COX1, DZIP1L, ZFP947, TRAFD1, ZFP949, PRKRIR, ZMAT1, PPARA, SH3RF1, PXDN, ZFP46, PPARG, GLI3, STAC3, GM14391, NUBP1, GM14393,	386	18	2.39E-08	HCCS, GDA, ITSN1, CIAPIN1, BRPF1, DDAH1, CRYAB, NME6, OGFOD1, PITPNM1, NME3, ZNHIT1, PELO, ZNHIT3, TMEM129, RPS27, CDADC1, ZC3H13, NPLOC4, KLF13, MAP2K2, TGFBR2, GAS6, HPCA, MZF1, CDC42BPA, CDC42BPB, ZFP385A, RNF187, MMP24, FBXL19, USP19, STAC2, SIK3, USP13, CAR14, ZSWIM6, ADNP, MMP17, ZSWIM7, VPS8, GNAL, TAF15, KSR1, PHYHD1, REPS2, FHL2, NECAB3, NECAB2, PLA2G12A, ILVBL, LIMD2, TCEA2, FEN1, GALNT14, BRD1, PPP1R10, TRIM25, SDHB, MNAT1, LRP1, CDH15, PDE2A, SDHC, MCFD2, NARFL, DGKZ, GNA12, MIPEP, ZFP787, ZFP580, PVALB, PDE4A, ZFP688, CTDSP2, RPS27A, HMGCL, PHRF1, ZFP692, ACTN4, MTA3, PDXP, STIM1, ACTN2, RNASEH2A, KDM2A, ZFP791, TESK1, ZFP511,

				P4HA3, NOS2, COL11A2, IHH, PRSS37, KCNMA1, ZFP422, ACO1, ZSWIM6, CYB5B, RBBP6, PJA2, RNF180, KSR2, ZFP866, ASH1L, GNAS, ZMYND11, NKD2, ZFP712, TH, FHL3, MYCBP2, DGKA, DGKB, MORC4, PPP2CA, ETFDH, PRKAA2, RBM26, LRSAM1, ZFP57, ATP1A3, ZFP119B, TRIM26, ATP1A1, TRIM24, SF3A2, CAPN2, PCK1, ZFP9, ZFP106, CYP4F18, SDHC, ZFP207, CHN1, NR5A1, CALM1, APOBEC3, GM5136, GNA11, LNX2, LMO7, COX5B, ZFP786, RNF141, CYP2J11, TIMM9, CTDSP2, ASPH, 2010111I01RIK, AMY1, ZFP692, KCND1, NUDT5, PDE4D, PDE4C, SUZ12, ZFP598, PDE5A, VSNL1, RNF138, COL1A1, CAR4, PPP5C, CAR7, ARFGAP1, CNBP, ASTN2, RIOK1, LIN28A, ITGAM, RBX1, VDR, PDE6C, ZFP760, RNF168, TRAF7, B4GALT6, RNF14, RNF13, TAF3, WHSC1, ITGA4, CACNA1S, GM14326, TRIM56, PHF3, GM14325,				ARFGAP1, ARFGAP2, TRAF2, CNBP, ME3, ELAC2, TIMP4, ZFP763, TIMP3, ECE2, RNF165, ADAM33, RNF169, RNF167, DDX41, B4GALT6, TRAF6, SDF4, B4GALT7, TRAF4, TRP53, B4GALT3, B4GALT2, TRIP4, EHMT1, AKAP8L, MYL12B, RFNG, VAV1, B3GAT3, EBF4, DOHH, POLD1, CARS2, HIVEP1, RPL37A, ABL2, CACNA1A, HDAC7, ZFP13, CYC1, ZKSCAN1, NDUFS7, TRIM8, HMOX1, ZCCHC9, ZFPL1, E4F1, CDK5RAP1, NDUFS2, KDM5B, ZCCHC2, ZFP553, TRPM7, IRF2BP2, TOPORS, ZFP652, PPM1F, ISCA2, PPM1K, ZFAND2B, CHFR, KDM6B, ACVR1, CYP2U1, PHLPP1, EGLN2, CABP5, TIMM13, CDH2, CDH5, DPF1, APLP1, CDH8, NUDT8, RNF128, NAIP5, GGPS1, ZFP524, ZFP523, EHD1, CHD4, VPS18, PDF, SLX1B, CYP20A1, ADI1, CADPS, RNF115, ZBED4, SMPD1, ZFP536, S100A3, S100A6, ALAD, ZFP637, QPCTL, ZFP335, SYT3, PRIM2, NT5C2, CYHR1, S100A1, YDJC, POLL, ZC3HC1, RPP21, ZFP467, NUDT11, PRDM8, UHRF2, PIAS4, SIAH1A, UBR5, DEAF1, MYO9B, CALR, ZFP457, RIMS1,

				SALL4, ZFP146, S100B, ITGA6, GM14322, FREM2, ZIC4, PRICKLE2, CYP2C38, AGTPBP1, MOB3C, PINK1, ZKSCAN1, DPH3, UQCRFS1, RHOU, TNIP2, IMPDH1, IMPDH2, ARL1, HMGCLL1, CYCS, RING1, MBNL2, COQ7, XRCC6BP1, ARL3, LAP3, RFWD2, ACVR2A, PPM1D, RFWD3, ZFP658, MDM2, UGP2, PEG3, AOC3, ING3, ZFP397, GALNT7, ADAMTS16, GLIS1, TIPARP, CYTB, BRSK1, NR3C1, ZFP661, CDH4, DROSHA, ADAT1, NPTX1, RASGRP1, ZFP664, PIKFYVE, TGM3, PLCD4, SCO1, ZBTB7B, INSM1, CYP2C70, WDFY1, ZFP386, CYP21A1, GM14410, TAB2, TAB3, SIRT3, GM14418, APIP, ALKBH3, ALKBH2, ALKBH5, ARAP1				TCF20, GALNS, NR1H3, ZFP346, KLF6, ZBTB48, IKZF4, CRIP1, OSGEP, SCD2, S100A16, HPCAL1, GDE1, SMYD2, ZFP445, ZFP746, U2AF1L4, ZBTB43, PPA1, CBLB, RNF7, ATP13A1, ATP2A3, RNF5, KLF2, PLA2G4B, TSNAX, ZMAT2, ZEB2, TRMT1, EFHD2, CGREF1, MBTD1, ELOF1, NT5M, NR2F6, ALOX12B, NEURL1B, NOS3, NEURL1A, AGAP3, ZBTB22, ZFP423, ZBTB20, ZCCHC10, SF1, HERC2, GMPR, RBBP6, ADO, ZFP865, RAB11FIP3, ZFP866, DNAJC24, ZFP869, ZFP961, THAP7, ZFP64, THAP4, ZFP414, THAP3, USP5, CETN2, ZFP316, PFAS, CYB561D2, CYB561D1, MORC3, TYW1, DTNB, ZFP410, BAZ2A, RBM22, MEX3B, ATP1A2, ATMIN, SOD2, BLVRA, PAPOLA, FYN, CYP4F16, ARSA, CALM2, APOBEC3, SLC6A1, CLYBL, FAHD1, GATA5, SLC25A23, TRIM44, RNF38, RABGEF1, PPP4C, ITPK1, RNF31, PICK1, TRIM41, ISCU, CPE, CHPF, MORC2A, RNF26, KALRN, ZC3H3, AMZ2, ITGB2, LMAN2, ZFAND1, RIOK2, RBX1, MYL9, PRR3, RNF10, STK38L, EWSR1, ACY3, ZMYM3, GALT, MUL1, UBOX5, TET2, LHPP,

								GM14326, GCM1, PRICKLE1, DNMT1, ASNA1, PHF8, GPATCH8, EPS15L1, CBFA2T3, PSPH, PDCD2, GSS, DGCR8, MT3, EGR1, SUOX, ELP3, WBSCR17, NRXN3, PFKP, ADIPOR2, ZFP322A, PRKCE, GZF1, NUCB1, MYT1L, DDR1, EYA1, DIS3L2, AOC2, ZFP367, FTL1, ZC4H2, NANOS1, GLRX2, RASGRP2, PLCD3, MUS81, SCNM1, PLCD1, ZFP292, CAMK2A, ENO1, DUS3L, DTX4, ZBTB7A, DTX1, SIRT4, SIRT7, PARK2, SPARC, HBA-A1, TDP2, ZC3H11A, ATP8A1, RNF41
**Zinc-finger**	123	8	1.92E-04	PNMA3, DZIP1, LNX2, ZNRF4, RP9, RORB, ZFP786, BTK, RNF141, ZFP930, U2AF1, ZRSR1, ZFP936, ZFP692, PLD6, RXRG, SUZ12, PRDM9, ZFP598, HNF4A, ZGLP1, RNF138, UNC13B, ARFGAP1, FUS, CNBP, ZCRB1, AKAP13, LIN28A, RBX1, PLAGL1, VDR, ZFP760, RNF168, TRAF7, RNF14, RNF13, AEBP2, TAF3, WHSC1, ZFP609, ZFP940, GM14326, CBLC, TRIM56, PHF3, GM14325, ZFP442, SALL4, RNF7, ZFP146, RAPSN,	187	9	1.07E-05	ZFP637, ZFP335, ZFP787, ZFP580, BRPF1, GATA5, TRIM44, RABGEF1, ZFP688, RNF38, CYHR1, RPS27A, RNF31, ZFP692, PHRF1, ZC3HC1, MTA3, TRIM41, ZFP467, PRDM8, UHRF2, PIAS4, KDM2A, UBR5, SIAH1A, ZFP791, MORC2A, ZNHIT1, RNF26, ZFP511, ZNHIT3, ZC3H3, ARFGAP1, DEAF1, TRAF2, ARFGAP2, CNBP, MYO9B, ZFP763, ZFAND1, RIMS1, ZFP457, RBX1, TMEM129, TCF20, RPS27, PRR3, RNF165, RNF169, RNF10, RNF167, TRAF6,

				GM14322, ZIC4, DZIP1L, ZFP947, ZFP949, TRAFD1, PRKRIR, ZMAT1, SH3RF1, PPARA, ZFP46, PPARG, ZKSCAN1, DPH3, GLI3, STAC3, GM14391, GM14393, TNIP2, ZFP422, ZSWIM6, RING1, MBNL2, RBBP6, RNF180, PJA2, RFWD2, KSR2, RFWD3, ZFP658, ZFP866, ASH1L, MDM2, PEG3, ZMYND11, ING3, ZFP397, TIPARP, ZFP712, GLIS1, FHL3, ZFP661, NR3C1, MYCBP2, DGKA, DGKB, MORC4, RASGRP1, PIKFYVE, ZFP664, RBM26, ZBTB7B, INSM1, LRSAM1, WDFY1, ZFP57, ZFP386, ZFP119B, GM14410, TRIM26, TRIM24, SF3A2, TAB2, TAB3, GM14418, ZFP9, ZFP106, CHN1, ZFP207, ARAP1, NR5A1				DDX41, TRAF4, EWSR1, NR1H3, ZFP346, IKZF4, ZC3H13, ZBTB48, KLF6, NPLOC4, TRIP4, KLF13, ZMYM3, AKAP8L, MUL1, SMYD2, UBOX5, ZFP445, VAV1, U2AF1L4, ZFP746, ZBTB43, GM14326, CBLB, RNF7, EBF4, RNF5, POLD1, MZF1, CDC42BPA, DNMT1, HIVEP1, RPL37A, KLF2, PHF8, GPATCH8, CDC42BPB, ZFP385A, ZFP13, ZMAT2, RNF187, TRMT1, ZKSCAN1, ZEB2, CBFA2T3, PDCD2, FBXL19, USP19, STAC2, MBTD1, TRIM8, ELOF1, NR2F6, ZCCHC9, ZFPL1, NEURL1B, E4F1, NEURL1A, KDM5B, AGAP3, USP13, ZBTB22, EGR1, ZFP423, ZCCHC10, ZBTB20, ZCCHC2, ZFP553, ZSWIM6, SF1, ADNP, ZSWIM7, IRF2BP2, ZFP322A, TOPORS, HERC2, PRKCE, RBBP6, GZF1, VPS8, ZFP652, MYT1L, ZFP865, ZFP866, TAF15, DNAJC24, ZFAND2B, ZFP869, KSR1, ZFP961, CHFR, ZFP367, THAP7, ZFP64, THAP4, THAP3, ZC4H2, ZFP414, USP5, NANOS1, FHL2, ZFP316, DPF1, MORC3, RASGRP2, RNF128, DTNB, ZFP410, ZFP524, ZFP523, SCNM1, ZFP292, TCEA2, BAZ2A, CHD4, DUS3L, RBM22, DTX4, ZBTB7A, BRD1,

								VPS18, DTX1, SLX1B, MEX3B, PPP1R10, TRIM25, PARK2, ATMIN, MNAT1, RNF115, ZBED4, DGKZ, ZC3H11A, ZFP536, RNF41
**Zinc**	**153**	**10**	**8.77E-04**	**APOBEC3, PNMA3, GM5136, DZIP1, LNX2, LMO7, ZNRF4, RP9, RORB, COX5B, ZFP786, BTK, RNF141, ZFP930, TIMM9, U2AF1, ZRSR1, 2010111I01RIK, ZFP936, ZFP692, CLCA1, KCND1, PLD6, RXRG, SUZ12, PRDM9, ZFP598, HNF4A, PDE5A, ZGLP1, RNF138, ADAMTS1, CAR4, UNC13B, CAR7, FUS, ARFGAP1, ERMP1, CNBP, AKAP13, ZCRB1, UBA5, LIN28A, RBX1, PLAGL1, VDR, ZFP760, ZMPSTE24, RNF168, TRAF7, RNF14, RNF13, AEBP2, BMP1, TAF3, AGBL4, WHSC1, AFG3L1, ZFP609, ZFP940, GM14326, CBLC, TRIM56, PHF3, GM14325, ZFP442, RNF7, SALL4, ZFP146, S100B, RAPSN, GM14322, ZIC4, DZIP1L, PRICKLE2, ZFP947, ZFP949, TRAFD1, PRKRIR, ZMAT1, SH3RF1, PPARA, ZFP46, AGTPBP1,**	**237**	**12**	**3.55E-05**	**S100A3, ALAD, GDA, ZFP637, QPCTL, ZFP335, BRPF1, DDAH1, CYHR1, S100A1, ZC3HC1, RPP21, CRYAB, ZFP467, PRDM8, UHRF2, PIAS4, UBR5, SIAH1A, ZNHIT1, ZNHIT3, DEAF1, MYO9B, CALR, ZFP457, RIMS1, TMEM129, TCF20, RPS27, CDADC1, NR1H3, ZFP346, IKZF4, ZC3H13, KLF6, ZBTB48, NPLOC4, CRIP1, KLF13, SMYD2, ZFP445, U2AF1L4, ZFP746, ZBTB43, CBLB, RNF7, RNF5, MZF1, CDC42BPA, KLF2, CDC42BPB, ZFP385A, ZMAT2, RNF187, ZEB2, TRMT1, MMP24, FBXL19, USP19, STAC2, MBTD1, ELOF1, NR2F6, NOS3, NEURL1B, NEURL1A, AGAP3, USP13, ZBTB22, ZFP423, ZCCHC10, ZBTB20, CAR14, SF1, ZSWIM6, ADNP, MMP17, ZSWIM7, HERC2, RBBP6, VPS8, ZFP865, ZFP866, TAF15, DNAJC24, ZFP869, KSR1, ZFP961, THAP7, ZFP64, THAP4, THAP3, ZFP414, USP5, FHL2, ZFP316, MORC3, DTNB, ZFP410, LIMD2, TCEA2, BAZ2A, RBM22,**

				MOB3C, PPARG, ZKSCAN1, DPH3, GLI3, STAC3, GM14391, GM14393, NOS2, TNIP2, IHH, ZFP422, ZSWIM6, RING1, MBNL2, RBBP6, LAP3, RFWD2, RNF180, PJA2, KSR2, RFWD3, ZFP866, ZFP658, ASH1L, MDM2, PEG3, ZMYND11, ING3, ZFP397, ADAMTS16, GLIS1, ZFP712, TIPARP, FHL3, ZFP661, NR3C1, MYCBP2, DROSHA, DGKA, ADAT1, DGKB, MORC4, RASGRP1, PIKFYVE, ZFP664, RBM26, ZBTB7B, INSM1, LRSAM1, WDFY1, ZFP57, ZFP386, ZFP119B, TRIM26, GM14410, TRIM24, SF3A2, TAB2, TAB3, SIRT3, GM14418, ZFP9, ZFP106, CHN1, ZFP207, APIP, ARAP1, NR5A1				BRD1, MEX3B, PPP1R10, TRIM25, ATMIN, BLVRA, MNAT1, DGKZ, APOBEC3, MIPEP, ZFP787, ZFP580, GATA5, TRIM44, RABGEF1, ZFP688, RNF38, RPS27A, RNF31, ZFP692, PHRF1, PICK1, MTA3, TRIM41, CPE, KDM2A, ZFP791, MORC2A, RNF26, ZFP511, ZC3H3, ARFGAP1, TRAF2, ARFGAP2, CNBP, ELAC2, AMZ2, TIMP4, ZFP763, TIMP3, ZFAND1, RBX1, ECE2, PRR3, SLC30A1, RNF165, RNF169, ADAM33, RNF10, SLC30A3, RNF167, TRAF6, DDX41, TRAF4, EWSR1, TRP53, EHMT1, TRIP4, ACY3, ZMYM3, AKAP8L, GALT, MUL1, UBOX5, TET2, VAV1, GM14326, GCM1, EBF4, PRICKLE1, POLD1, DNMT1, CARS2, HIVEP1, RPL37A, ASNA1, PHF8, GPATCH8, HDAC7, ZFP13, ZKSCAN1, CBFA2T3, PDCD2, TRIM8, ZFPL1, ZCCHC9, E4F1, GPC1, KDM5B, MT3, EGR1, ZCCHC2, ZFP553, TRPM7, ADIPOR2, IRF2BP2, ZFP322A, TOPORS, PRKCE, GZF1, ZFP652, MYT1L, ZFAND2B, CHFR, KDM6B, ZFP367, ZC4H2, NANOS1, TIMM13, APLP1, DPF1, RASGRP2, NAIP5,

								**RNF128, ZFP524, ZFP523, SCNM1, ZFP292, CHD4, DUS3L, DTX4, ZBTB7A, VPS18, DTX1, SLX1B, SIRT4, SIRT7, PARK2, RNF115, ZBED4, SMPD1, ZC3H11A, ZFP536, RNF41**

[1]Huang, D. W.; Sherman, B. T.; Lempicki, R. A. Systematic and integrative analysis of large gene lists using DAVID Bioinformatics Resources. *Nature Protoc.* **2009,** *4*, 44-57.
